# LINC00052 regulates the expression of NTRK3 by miR-128 and miR-485-3p to strengthen HCC cells invasion and migration

**DOI:** 10.18632/oncotarget.10250

**Published:** 2016-06-23

**Authors:** Dongmei Xiong, Yanrui Sheng, Shijia Ding, Juan Chen, Xixi Tan, Tao Zeng, Dongdong Qin, Liying Zhu, Ailong Huang, Hua Tang

**Affiliations:** ^1^ Key Laboratory of Molecular Biology for Infectious Diseases (Ministry of Education), Institute for Viral Hepatitis, Department of Infectious Diseases, The Second Affiliated Hospital, Chongqing Medical University, Chongqing, China; ^2^ Collaborative Innovation Center for Diagnosis and Treatment of Infectious Diseases, Zhejiang University, Hangzhou, China; ^3^ Department of Clinical Laboratory, Jining No.1 People's Hospital, Jining, China; ^4^ Key Laboratory of Clinical Laboratory Diagnostics (Ministry of Education), College of Laboratory Medicine, Chongqing Medical University, Chongqing, China

**Keywords:** HCC, LINC00052, NTRK3, invasion, migration

## Abstract

Long non-coding RNAs (LncRNAs) are a group of RNAs that are more than 200 nt in length but cannot encode proteins. Accumulating evidences showed that abnormal LncRNA expressions are highly involved in many kinds of tumor. By using gene trap methods which could knockdown gene expression to find important genes, we found one LncRNA which called intergenic non-protein coding RNA 52 (LINC00052) has the ability to inhibit invasion and migration of hepatocarcinoma cells. We found that invasion, migration and proliferation abilities in SMMC7721 cell were inhibited after up-expressing LINC00052. We identified that NTRK3 was the target gene of LINC00052. Down-expression of NTRK3 could increase SMMC7721 cell invasion, migration and proliferation. Meanwhile, we discovered that LINC00052 could regulate NTRK3 expression by forming complementary base pairing with miR-128 and miR-485-3p to reduce the luciferase activity of NTRK3 3′UTR. These results reveal a new mechanism for understanding hepatocarcinoma cells invasion and migration.

## INTRODUCTION

Hepatocellular carcinoma (HCC) is the fifth most common solid tumor and the third leading cause of cancer-related deaths worldwide [1]. Most cases of HCC are attributed to chronic infection with either hepatitis virus B or C [2]. Unfortunately, HCC patients remain poor prognosis and high recurrence rate despite recent advances in surgical resection and medical treatment [3].

Invasion and metastasis of cancer cells is a complex, multi-step biology process. In the biology process, the tumor cells break away from the original site of the occurrence length, invade and migrate to the paracarcinoma tissue and distant tissue, which involves tumor cells pass through the extracellular matrix barriers, basement membrane of blood vessel walls and other processes. Invasion and metastasis abilities of cancer cells are closely associated with the abilities that cancer cells produce proteolytic degradation of extracellular matrix and basement membrane of the vessel wall [4]. So far at least three categories of metastasis genes have been proposed to facilitate the multistep metastatic cascade: (1) “initiation” genes that facilitate detachment (e.g., CDH2 (encodes N-Cadherin) and TWIST), extracellular matrix degradation (e.g., MMPs) or angiogenesis (e.g., VEGF); (2) “progression” genes (e.g., PTGS2 (encodes COX-2) and MMP-1) that regulate extravasation of circulating tumor cells and are involved in metastatic colonisation; (3) “virulence” genes (e.g., IL6 and TNFα), which promote survival in circulation, and/or provide a proliferative advantage in the distant microenvironment. Apart from these metastasis-promoting genes, there is a well-distinguished class of metastasis “suppressor” genes that represses tumor cell dissemination without any effect on primary tumor growth, including KAI-1, BRSM1, and NME1 [5].

In order to find new genes which play a role in HCC metastasis, we used gene trapping technique to inhibit gene expression. With the help of randomly inserted mutation libraries developed by gene trapping technique, a lot of genes with unknown functions have been discovered and characterized [6]. In our experiment, the gene trapping vector PU21 [7] was transected into SMMC7721 cells and monoclonal cell lines were selected and obtained by using G418. The abilities of invasion and migration of these cell lines were further screened by using transwell assay. After that, gene cloning and gene sequencing were done to identify which gene has been trapped. Fortunately, we found that in A554 cell line, the gene trapped by PU21 vector was LINC00052.

LncRNAs are important signaling molecules of life which were discovered in recent years. Genome-wide transcriptomic studies have shown that the mammalian genome is abundantly transcribe and at least 80% of this transcription is exclusively associated with long non-coding RNAs (LncRNAs) [8]. LncRNAs have frequently been disregarded as artifacts of chromatin remodeling or transcriptional ‘noise’, and are frequently delimited long (generally > 2 and some > 100 kb), spliced and contain canonical polyadenylation signals. Long ncRNAs play important roles in epigenetic regulation of protein-coding gene expression, such as Hox gene [9]. Moreover, the dysregulation of LncRNAs appears to be a primary feature of many complex human diseases, including leukaemia, colon cancer, prostate cancer, breast cancer, hepatocellular carcinoma, psoriasis, ischaemic heart disease, Alzheimer's disease and spino-cerebellarataxia type 8 [9].

We found LINC00052 through gene trapping technique and found its ability to regulate invasion and migration in HCC cells. However, how LINC00052 to affect HCC cells invasion and migration, has not being reported. Here, we reported that LINC00052 could inhibit HCC cells invasion and migration through complementing with miR-128 and miR-485-3p, both of them can regulate NTRK3 [10](neurotrophic tyrosine kinase receptor, type 3) gene expression. And NTRK3 can affect HCC cells invasion and migration. Our results elucidated that LINC00052 might have a tumor suppressor function in HCC and could be a novel potential target for therapy of HCC.

## RESULTS

### LINC00052 was trapped in A554 cell line

**Figure 1:**
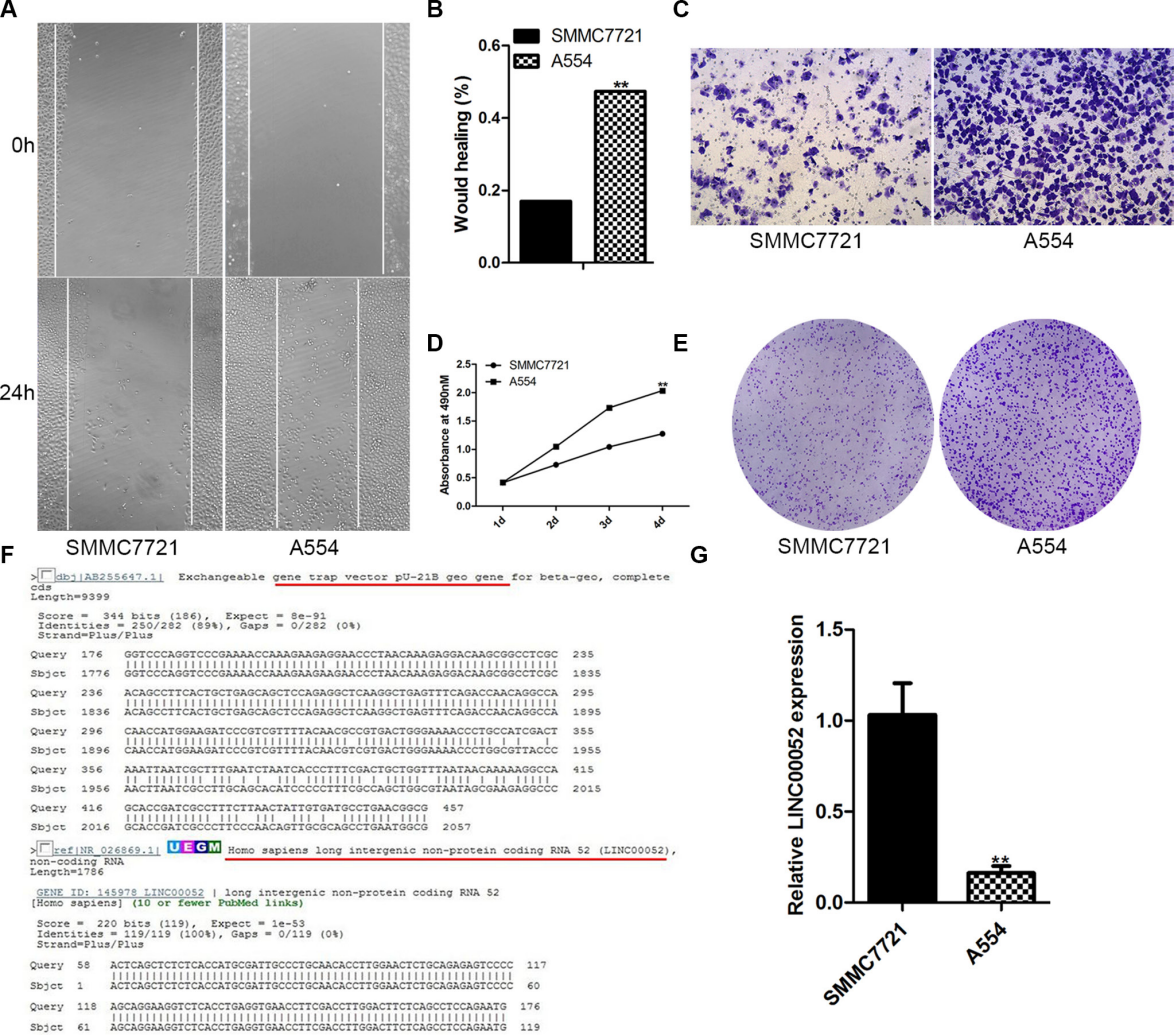
LINC00052 gene was trapped by PU21 in A554 cell line (**A**, **B**) Wound healing assay, SMMC77212 cells as a control. (**C**) Transwell assay, SMMC77212 cells as a control. (**D**, **E**) Cell proliferations assay, SMMC77212 cells as a control. (**F**) 5′RACE result showed that LINC00052 gene was trapped by PU21 vector in A554 cell line. (**G**) LINC00052 expressions were detected with real-time PCR in A554 cells, SMMC77212 cells as a control.

For finding new gene which has a function in HCC cells invasion and migration, gene trapping vector PU21 were transfected into SMMC77221 hepatoma cells and selected by G418. Lots of cell colonies were set up and then cultured into cell lines. These cell lines were further screened with transwell assays and wound healing assays. A cell line, A554, had a stronger migration (Figure 1A, 1B), invasion (Figure 1C) and proliferation (Figure 1D, 1E) ability comparison with SMMC7721 cells. RACE result showed that the gene trapped by PU21 in A554 cell line was LINC00052 (Figure 1F). Furthermore, real-time PCR demonstrated that the expression of LINC00052 was inhibited by gene trapping (Figure 1G). These data suggested that LINC00052 might be able to influence HCC cells invasion and migration.

### Analysis the expression of LINC00052 in HCC tissue and HCC cell lines

**Figure 2:**
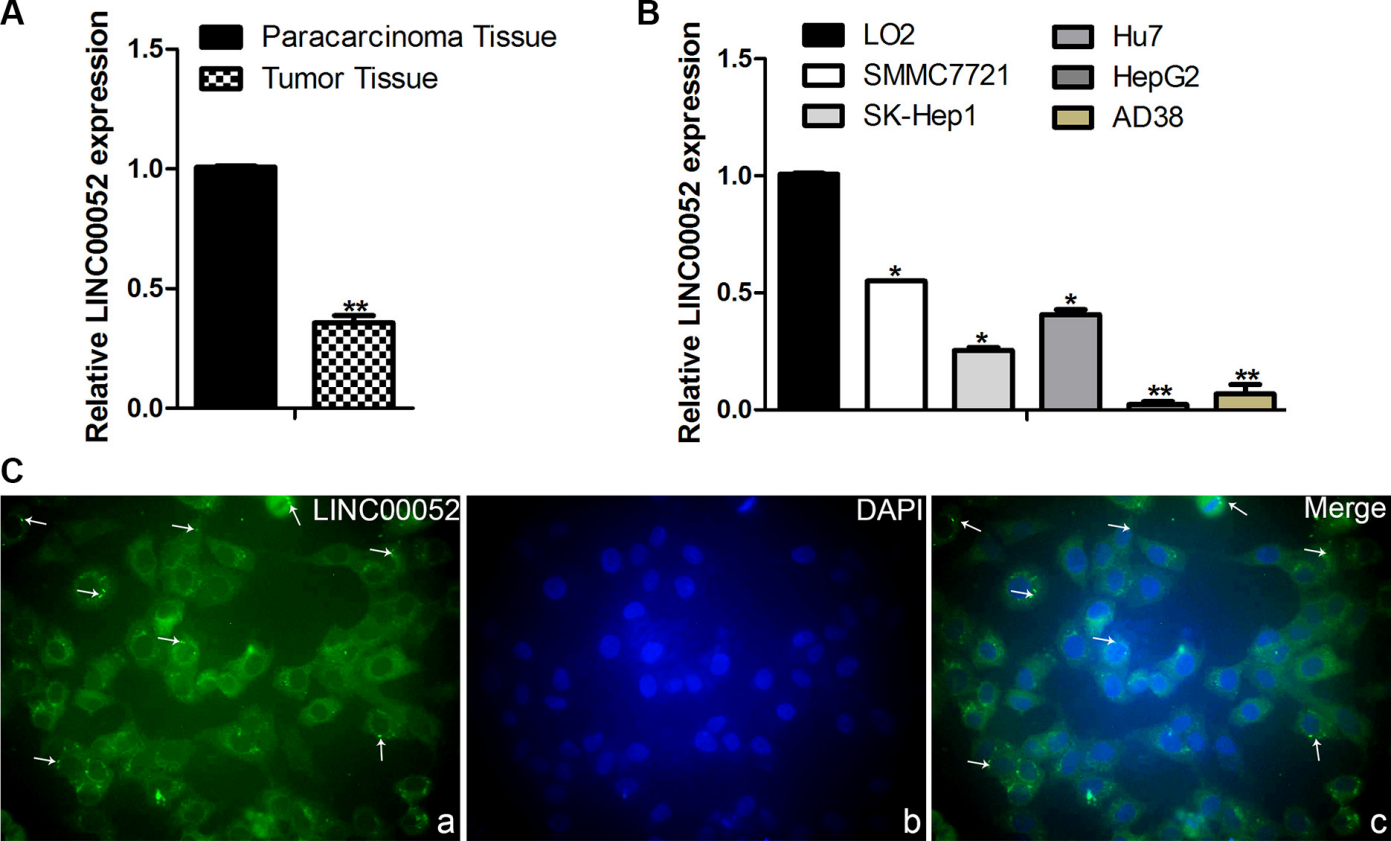
Analysis the expression of LINC00052 in HCC tissue and HCC cell lines (**A**) The expressions of LINC00052 in HCC tissue, the paracarcinoma tissue as a control. (**B**) The expressions of LINC00052 in HCC cell lines, LO2 cells as a control. (**C**) The cellular localization of LINC00052 was identified with FISH. a: LINC00052 RNA probe, b: DAPI Staining, c: Merge. Original magnification: 400 ×.

In order to detect the expression of LINC00052 in HCC tissue and HCC cell lines, real time PCR test was used. Compared with the paracarcinoma tissue, the expression of LINC00052 was lower in HCC tissue (Figure 2A), and compared with the normal liver cell line LO2, LINC00052 were down-expressed in the five HCC cell lines: SMMC7721, SK-Hep1, Hu7, HepG2 and AD38 (Figure 2B). Furthermore, LIN00052 expressed in the cytoplasm was indentified with FISH analysis (Figure 2C)

### LINC00052 could influence HCC cells migration and proliferation

**Figure 3:**
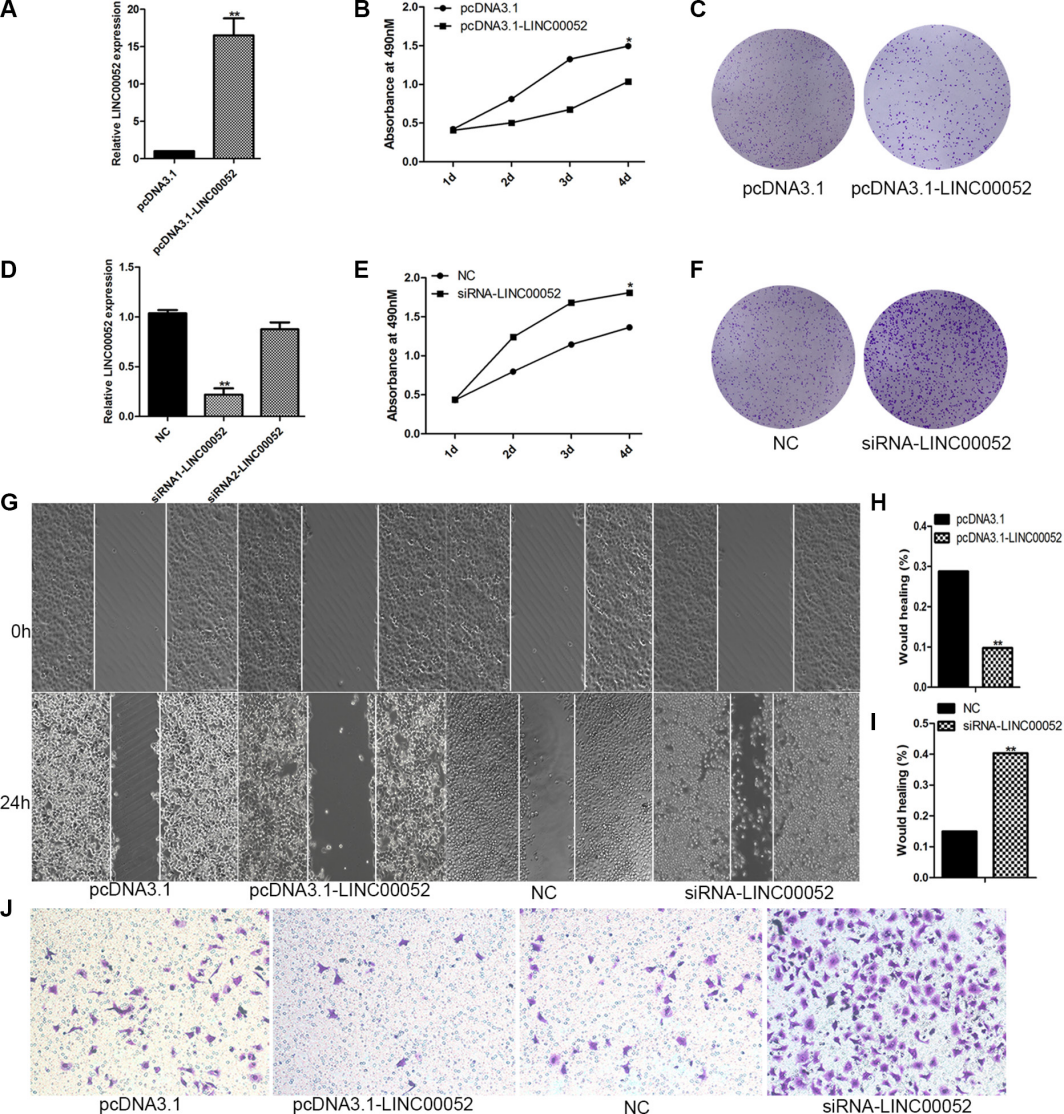
LINC00052 influence HCC cells proliferation, migration (**A**) Over expression of LINC00052 was confirmed with real-time PCR. (**B**, **C**) Cell proliferations were verified by MTS and Colony formation assay when LINC00052 expressions were over expressed. (**D**) Inhibition of LINC00052 with siRNA was confirmed. (**E**, **F**) Cell proliferations were verified by MTS and Colony formation assay when LINC00052 expressions were inhibited with siRNA. (**G**, **H**, **I**) Cell migrations were detected by using wound healing assay when SMMC77221 cells over or down expression of LINC00052. (**J**) Cell invasions were detected by using transwell assay when SMMC77221 cells over or down expression of LINC00052.

In order to test whether LINC00052 could really influence HCC cells invasion, migration and proliferation, we generated pcDNA3.1-LINC00052 plasmid to over-express LINC00052, and synthesized siRNA to inhibit expression of LINC00052. Firstly, the efficiency for over-expression or down-expression of LINC00052 was confirmed by using qPCR (Figure 3A, 3D). The siRNA1 targeting LINC00052 was used for the subsequent experiment due to its high interference efficiency. Then pcDNA3.1-LINC00052 or siRNA were transfected into SMMC7721 cells, and cell invasion, migration and proliferation were detected by using transwell assay, wound healing assay and MTS test and colony formation assay. We found that cell proliferation abilities were inhibited in cells over expressing LINC00052 (Figure 3B, 3C). Conversely, knockdown LINC00052 increased cells proliferation abilities (Figure 3E, 3F). Meanwhile, cell migration and invasion abilities were inhibited in cells over expressing LINC00052, but induced in cells down expressing LINC00052 (Figure 3G–3J). These results showed that LINC00052 indeed regulated HCC cells invasion, migration and proliferation abilities.

### NTRK3 might be a target gene of LINC00052

**Figure 4:**
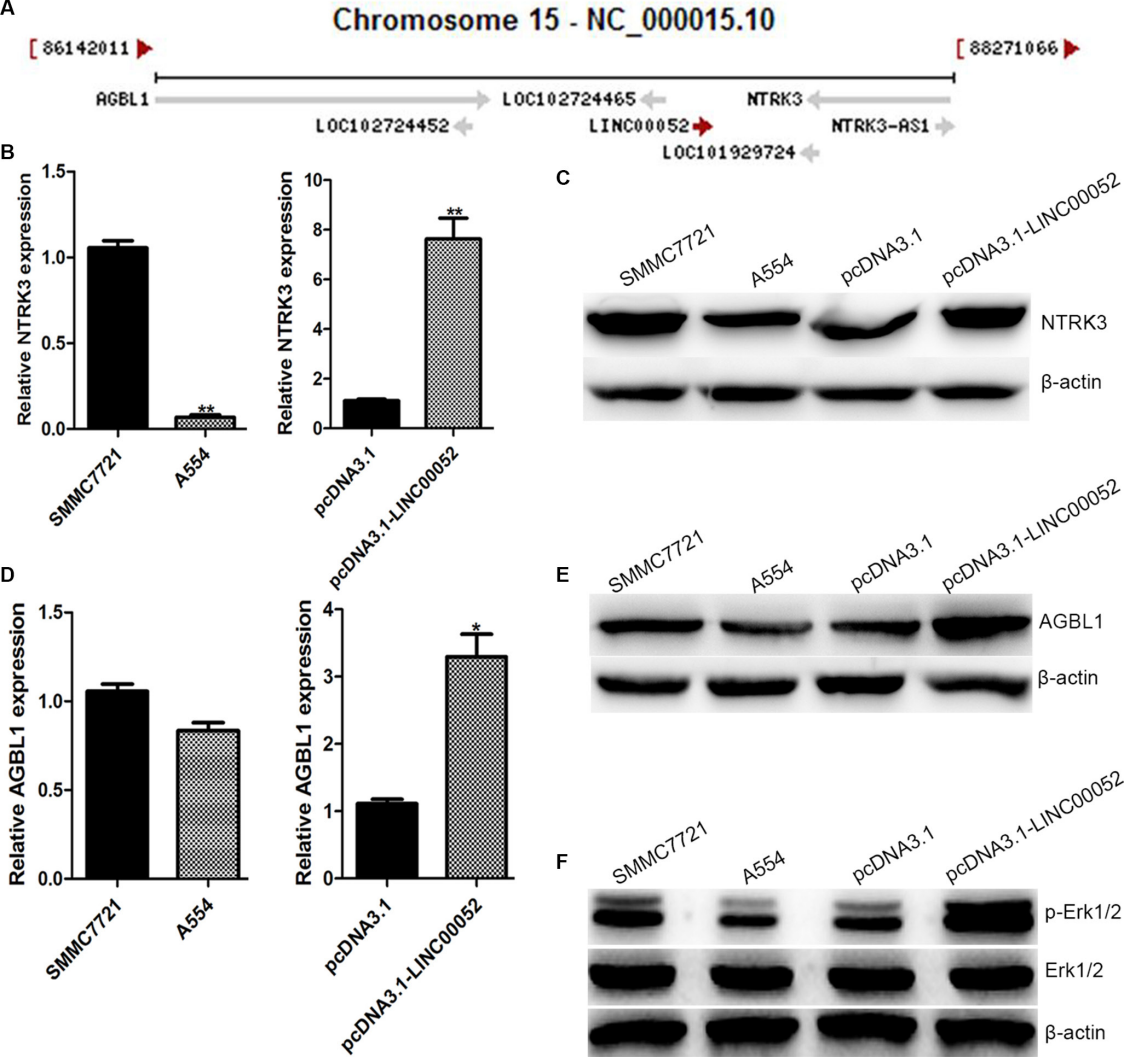
NTRK3 was a target gene of LINC00052 (**A**) LINC00052 was located between AGBL1 and NTRK3 on chromosome 15. (**B**) Expressions of NTRK3 in Low LINC00052 expression (A554 cells) and High LINC00052 expression (pcDNA3.1-LINC00052 cells) cells were detected with real-time PCR and western blotting (**C**). (**D**) Expressions of AGBL1 in Low LINC00052 expression (A554 cells) and High LINC00052 expression (pcDNA3.1-LINC00052 cells) cells were detected with real-time PCR and Western blotting (**E**). (**F**) Expressions of p-Erk1/2 in Low LINC00052 expression (A554 cells) and High LINC00052 expression (pcDNA3.1-LINC00052 cells) cells were detected with Western blotting.

To explore the possible mechanisms how LINC00052 affected HCC cells invasion and migration, LINC00052 target genes were investigated. It is reported that LncRNAs could perform their functions by interacting its nearby genes [11]. We found that LINC00052 was located between ATP/GTP binding protein-like1 (AGBL1) and NTRK3 on chromosome 15 (Figure 4A). These two gene expressions were checked in A554 cells or cells over expressing LINC00052 (pcDNA3.1-LINC00052 cells). Real-time PCR and Western blot results showed that both NTRK3 and AGBL1 expressions had significant changes both at RNA and protein level (Figure 4B–4E). This result suggested that NTRK3 and AGBL1 might be target genes of LINC00052. However, only NTRK3 was studied in this research, AGBL1 was detected in other study. Furthermore, we also analyzed NTRK3 down signaling molecular p-Erk1/2 [12] expression in A554 cells and over expression LINC00052 cells. We found that p-Erk1/2 was down-expressed after down-expression LINC00052 as NTRK3. And p-Erk1/2 was up-expressed after up-expression LINC00052 as NTRK3 (Figure 4F).

### LINC00052 modulated NTRK3 expression by interacting with miR-128 and miR-485-3p

**Figure 5:**
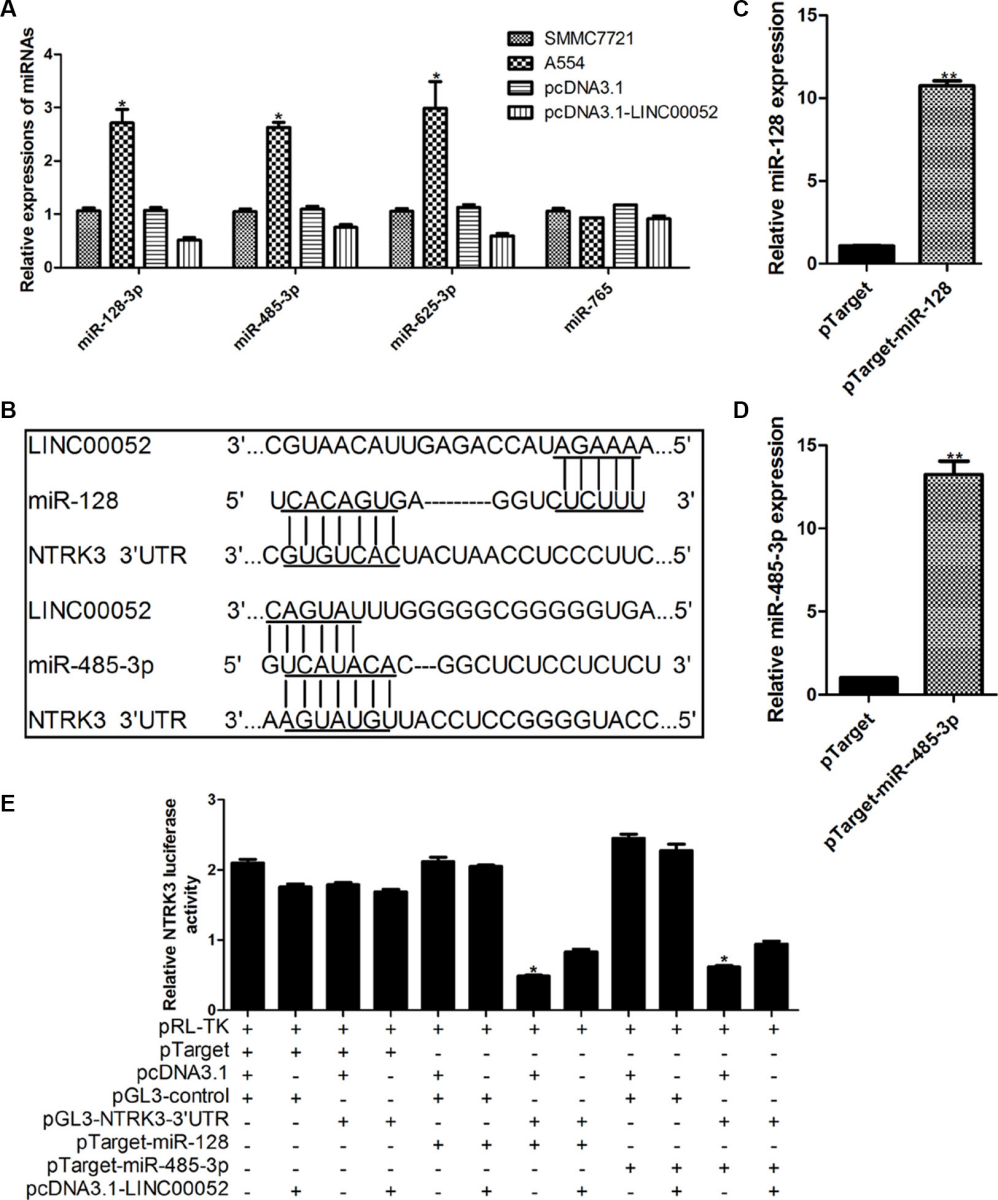
LINC00052 modulated NTRK3 expression by interacting with miR-128 and miR-485-3p (**A**) Expressions of microRNAs which involved in regulating MTRK3 were checked in low LINC00052 expression (A554 cells) and high LINC00052 expression (pcDNA3.1-LINC00052 cells) cells. (**B**) miR-128 and miR-485-3p could form complementary bases with LINC00052 and NTRK3 3′UTR. (**C**), (**D**) Over expression miR-128 and miR-485-3p were constructed and verified by real-time PCR. (**E**) Luciferase activity of NTRK3 3′UTR were detected after cotransfected with pTarget-miR-128, pTarget miR-485-3p and pcDNA3.1-LINC00052. pTarget, pcDNA3.1 and pGL3-control were used as controls.

For finding molecular mechanism which LINC00052 mediated NTRK3 expression, we carried out several experiments to identify whether LINC00052 could interact with some microRNAs which regulate NTRK3. Margarita et al. has reported that significant reduction of the luciferase activity of NTRK3 3′UTR was observed after cotransfected with miR-128, miR-485-3p, miR-509, miR-625, and miR-765 when compared with two different control mimics [10]. Therefore we supposed whether LINC00052 could influence NTRK3 expression by forming complementary base pairing with these microRNAs. qPCR results showed that the expressions of miR-128, miR-485-3p and miR-625 could be influenced by LINC00052 (Figure 5A). However, we found that only miR-128 and miR-485-3p formed complementary bases with LINC00052 and NTRK3 3′UTR simultaneously (Figure 5B). Then, the plasmids expressing miR-128 and miR-485-3p were transfected in HCC cells, to investigate whether miR-128 and miR-485-3p could reduce luciferase activity of NTRK3 3′UTR (Figure 5C, 5D). Results showed luciferase activity of NTRK3 3′UTR was inhibited when miR-128 and miR-485-3p were overexpressed. However, this inhibition could be partially rescued when LINC00052 was existed (Figure 5E). These results implied that LINC00052 manipulated NTRK3 expression by forming complementary base pairing with miR-128 and miR-485-3p.

### NTRK3 was closely related with cells proliferation, invasion and migration

**Figure 6:**
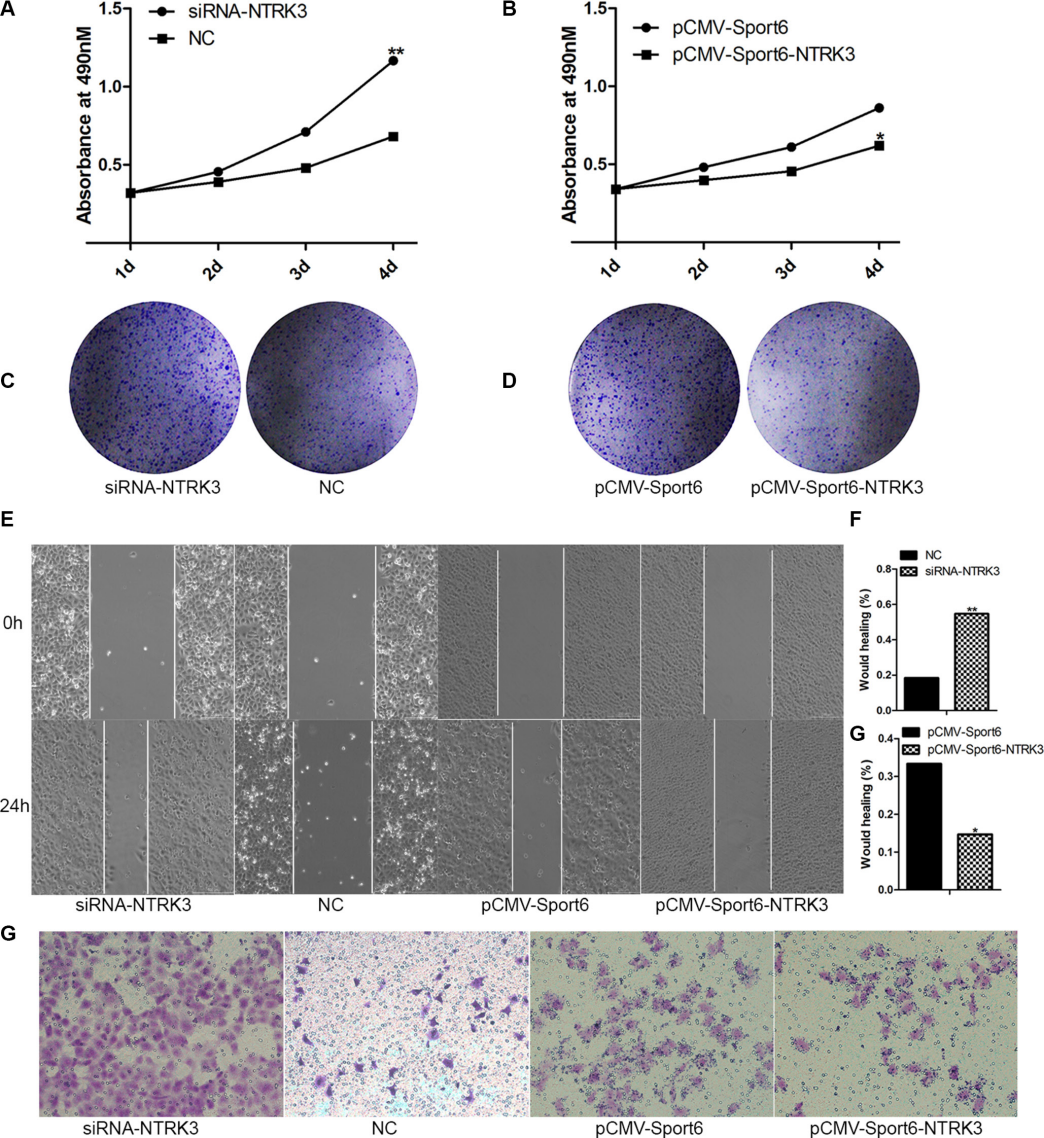
Analysis of NTRK3 functions in cells proliferation, invasion and migration (**A**) Cell proliferations were verified when NTRK3 expression were inhibited with siRNA or when NTRK3 expression were over expressed (**B**). (**C**), (**D**) Colony formation assay. (**E**, **F**, **G**) Wound healing assay. (**F**) Transwell assay. NC and pCMV-Sport6 were used as controls.

In order to discover whether NTRK3 was related with cells invasion and migration abilities or not, plasmid expressing NTRK3 (pCMV-Sport6-NTRK3) and siRNA targeting NTRK3 were constructed and synthesized (Supplementary Figure S1). Interestingly, we found, cells proliferation and colony formation were increased in NTRK3-depleted cells compared with the control cells (Figure 6A, 6C). Conversely, cells proliferation and colony formation were reduced when NTRK3 was over-expressed (Figure 6B, 6D). Meanwhile, cells invasion and migration were increased in NRTK3-depleted cells or decreased in NRTK3-overexpressing cells (Figure 6E–6H). In order to fully confirm functions of NTRK3, the same experiments were repeated in another HCC cells, SK-Hep1 cells. The similar results were obtained in the SK-Hep1 cells (Supplementary Figure S2). These results showed that NTRK3 played a role in regulating cancer cells proliferation, invasion and migration.

### LINC00052 influenced tumor growth *in vivo*

**Figure 7:**
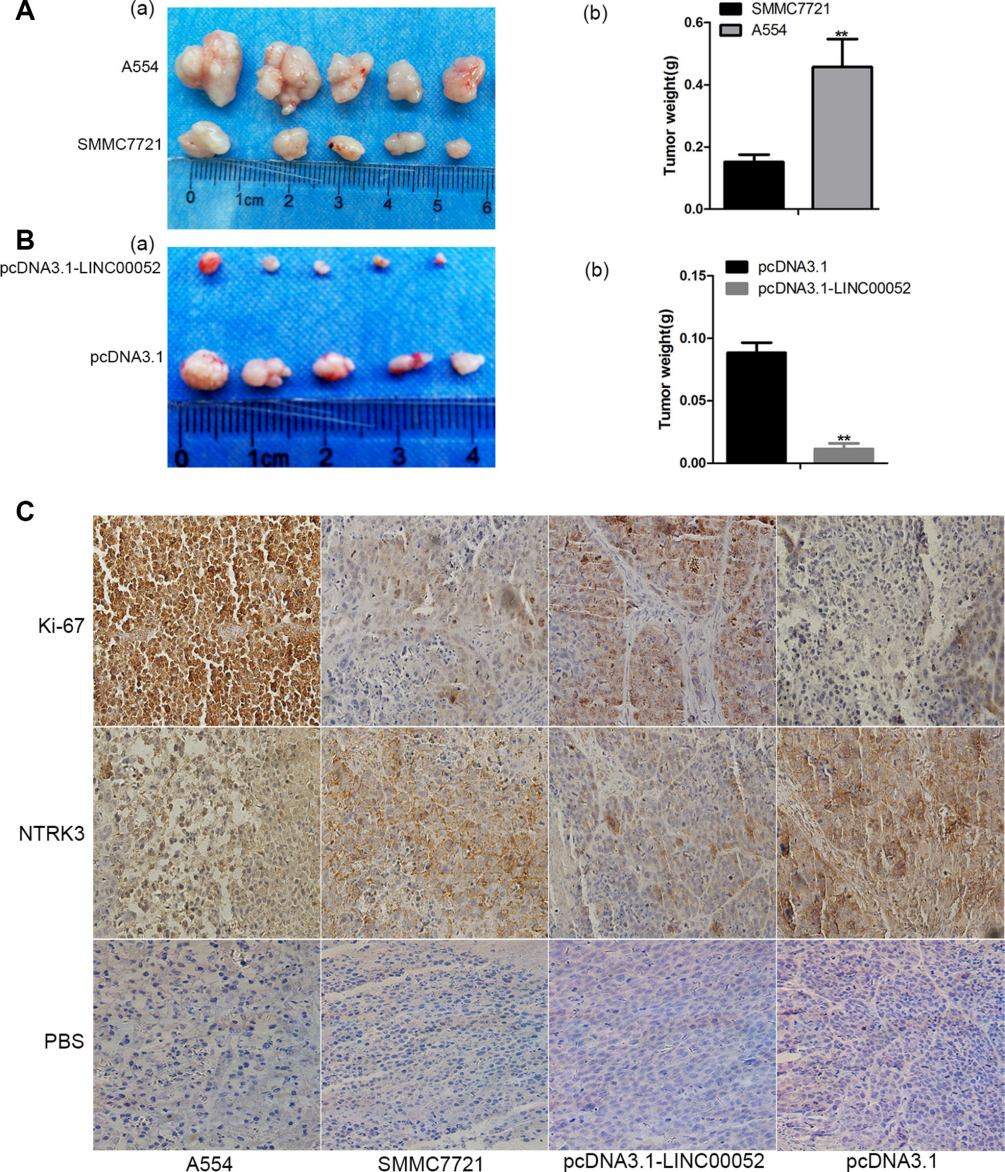
Role of LINC00052 in tumor development *in vivo* (**A**) Tumor sizes were recorded (a) and weighed (b) after A554 cells were injected (low LINC00052 expression), SMMC7721 cells as a control. (**B**) Tumor sizes were recorded (a) and weighed (b) after LINC00052 stable over expression cells were injected, pcDNA3.1 stable cells as a control. (**C**) Ki-67 and NTRK3 immunostaining in transplanted tumors. PBS used as a control. Original magnification: 400 ×.

To investigate the functional role of LINC00052 in tumor development *in vivo*, cells stably over-expressing LINC00052 (pcDNA3.1-LINC00052), or cells down-expressing LINC00052 (A554) and their control cells were injected into buttock of nude mice, respectively. Four weeks later, nude mice were sacrificed and xenografts were weighed. We found that the tumor size formed by LINC00052 knockdown cells showed was bigger and heavier compared with the control group (Figure 7A). In contrast, the xenografts formed by cells over-expressing Linc00052 were smaller and lighter than the control group (Figure 7B). Meanwhile, the immunohistochemistry results found that tumor formed by LINC00052-deplted cells showed increased expression of proliferation marker Ki-67 and decreased expression of NTRK3. In contrast, decreased expression of proliferation marker Ki-67 and increased expression of NTRK3 were observed in tumors formed by LINC00052-overexpressing cells (Figure 7C). Taken these results together, we concluded that LINC00052 could influence tumor proliferation *in vivo*.

### LINC00052 influenced tumor migration *in vivo*

**Figure 8:**
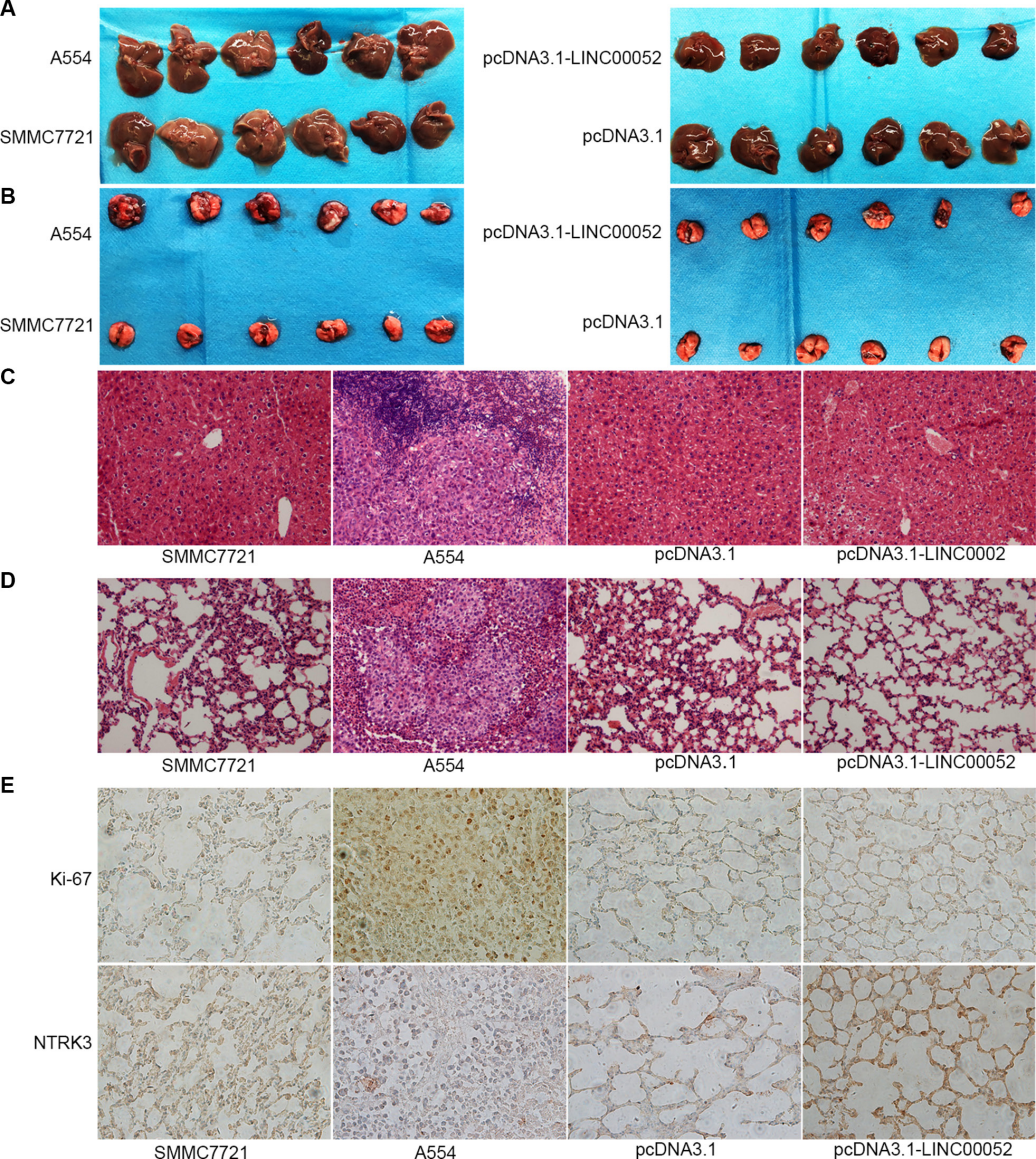
Role of LINC00052 in tumor migration *in vivo* (**A**) Tumor cell migrations in liver tissues. (**B**) Tumor cell migrations in lung tissues. (**C**) Tumor cell migrations in liver tissues were detected by HE staining assay. (**D**) Tumor cell migrations in lung tissues were detected by HE staining assay. (**E**) Ki-67 and NTRK3 immunostaining in lung tissues. Original magnification: 400 ×.

To investigate the functional role of LINC00052 in tumor migration potential *in vivo*, cells stably over-expressing LINC00052 (pcDNA3.1-LINC00052), or cells down-expressing LINC00052 (A554) and their control cells were injected into liver of nude mice, respectively. 24 days later, nude mice were sacrificed and the migration potential were analyzed. We found that in A554 group, some metastatic lesions were found in liver and especially in lung tissue compared with SMMC7721 group, but in pcDNA3.1-LINC00052 group, no metastatic lesions were found in liver and lung tissue compared with pcDNA3.1 group (Figure 8A, 8B). Also tumor cell migration in liver and lung tissues were detected by HE staining assay. The results showed that in A554 group, a large number of lymphocytes were found in liver and lung tissue compared with SMMC7721 group, but in pcDNA3.1-LINC00052 group, no lymphocytes were found in liver and lung tissue compared with pcDNA3.1 group (Figure 8C, 8D). Furthermore, the lung immunohistochemistry results revealed that increased expression of proliferation marker Ki-67 and decreased expression of NTRK3 in LINC00052-deplted group. In contrast, decreased expression of proliferation marker Ki-67 and increased expression of NTRK3 were observed in LINC00052-overexpressing group (Figure 8E). Taken these results together, we concluded that LINC00052 could influence tumor migration *in vivo*.

### NTRK3 was down-expressed in human liver cancer tissue

**Figure 9:**
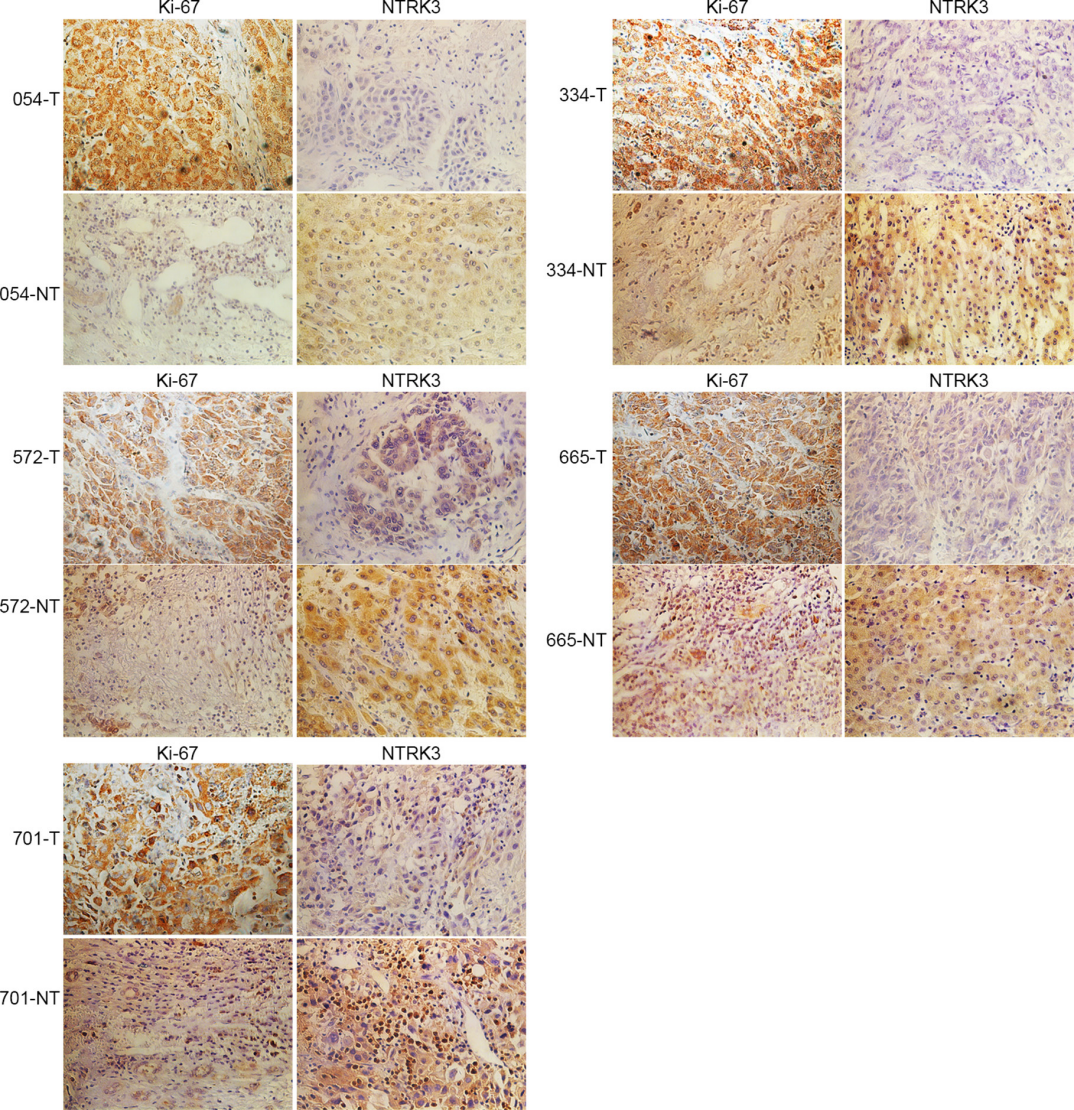
NTRK3 is down-regulated in human HCC tissue Human HCC tissue and paracarcinoma tissue sections were immunohistochemistry staining with Ki-67 and NTRK3 (magnification: 400 ×).

In order to detect the expression of the target gene NTRK3 in clinical specimen, liver cancer tissues and paracarcinoma tissues were used to perform immunochemistry study. Our results showed that the expressions of NTRK3 were down-expressed in human liver cancer tissues compared with paracarcinoma tissues (Figure 9).

## DISCUSSION

LncRNAs range from approximately 200 nt to over 100 kb in length, and play important functions in the life process in different ways. Studies have shown that LncRNAs participate in a wide variety of molecular genetics and cellular processes, including chromosomal dosage compensation, control of imprinting, chromatin modification, chromatin structure, transcription, splicing, translation and cellular differentiation [13]. Meanwhile, many diseases have been reported to be related with LncRNAs. For example, loss of imprinting at the H19 locus resulted in high H19 expression in cancers of the esophagus, colon, liver, bladder and with hepatic metastases [14–16]. Now, LncRNAs were regarded to perform their functions in the following aspects: transcriptional interference, chromatin remodeling and histone modifications, modulating alternative splicing patterns, generating endo-siRNAs, modulating protein activity, structural or organizational role, altering protein localization, and being small RNA precursor [17].

In our study, we discovered LINC00052 through Gene trap technology and we found that LINC00052 expressed in the cytoplasm and could affect hepatoma cells invasion and migration. We identified that LINC00052 suppression increased the invasion and migration in HCC SMMC7721 cells, while the invasion and migration of were reduced in SMMC7721 cells overexpressing LINC00052. In order to investigate the mechanism of LINC00052 influencing cells invasion and migration, NTRK3 was found to be the target gene of LINC00052 based on the location on the chromosome of LINC00052.

NTRK3, also known as TrKc, is one kind of Neurotrophin receptor. Neurotrophins receptors are a family of growth factors that play important roles in the nervous system and are composed of four members: nerve growth factor (NGF), brain-derived neurotrophic factor (BDNF), neurotrophin-3 (NT3), and neurotrophin-4/5 (NT4/5). Each member binds with high affinity to a specific neurotrophic tyrosine kinase (NTRK) receptor, and NTRK3 is the strongest affinity ligand of neurotrophin-3 [18]. Six different isoforms of the NTRK3 receptor have been identified. Isoforms of NTRK3, which are generated by alternative splicing of the intracellular domain (truncations or insertions), specifically respond to NT-3 [19]. Functionally, distinct NTRK3 isoforms may modulate signal transduction pathways by forming heterodimers with full-length receptors or by competitive ligand binding. Activation of truncated NTRK3 receptors inhibits full-length NTRK3 receptors and function as dominant-negative receptors [20, 21]. Studies have showed that NTRK3 play important roles in the nervous system, not only in normal life activities but also in diseases. However, NTRK3 has also been found in several nonneural cell types diseases (medullary thyroid carcinoma, breast cancer, colon cancer, lung cancer, liver cancer, prostate cancer) and may also play a crucial role in the initiation, progression, and migration of many tumors in human [22–24]. However, some studies show that NTRK3 is a potential tumor suppressor gene in colorectal cancer [12, 25] and is also highly expressed in good-prognosis neuroblastomas as well as medulloblastomas [26, 27], but NTRK3 works as a oncogene in breast tumor [28]. These studies show that NTRK3 has different roles in different cancers. Even if in the same cancer in different situations, such as stages, sex, ages and so on, NTRK3 has different roles. In our study, we found that NTRK3 was down-expressed in HCC cells with high invasion and migration, while up-expressed in HCC cells with low invasion and migration. This means that NTRK3 inhibited HCC cells invasion and migration, and it might work as a tumor suppressor in HCC.

In order to understand how LINC00052 regulate the expression of NTEK3, we set out to find a “bridge” between LINC00052 and NTEK3. It was reported that NTRK3 works with microRNAs to control proliferation of human neuroblastoma cells [29] and NTRK3 also is a susceptibility factor for Anxiety Disorders after interplaying with microRNAs [10]. Meanwhile, we found that one way of LncRNAs to exert biological functions was regulating the expression of microRNAs [30, 31]. So we supposed that whether LINC00052 could regulate the expression of NTRK3 by some microRNAs. Margarita et al. [10] found that the luciferase activity of the wild-type 3′UTR of the truncated isoform of NTRK3 was significantly downregulated by several microRNAs in HeLa cells. In our study, we made sequence analysis between LINC00052 and the five microRNAs and found that only miR-128 and miR-485-3p could form complementary base with LINC00052. Our results further found LINC00052 could influence the expressions of miR-128 and miR-485-3p. Meanwhile, miR-128 and miR-485-3p also could reduce the luciferase activities of the wild-type 3′UTR of the truncated isoform of NTRK3 in HCC cells. These results proved that LINC00052 could regulate the expression of NTRK3 by some microRNAs indeed.

More importantly, animal experiments showed that tumor formed by A554 cells increase tumor size and weight, and accompanied by decreased expression of NTRK3 and increased expression of Ki-67. We found that NTRK3 were down-regulating in liver cancer patient specimens compared with paracancerous specimens.

Taken together, our data showed that LINC00052 could regulate the expression of NTRK3 by miR-128 and miR-485-3p to influence the invasion and migration of HCC cells. These results suggest that LINC00052 may have potentially diagnostic and therapeutic value for HCC in the future.

## MATERIALS AND METHODS

### 5′ RACE PCR

5′ RACE PCR was performed by using 5′-Full RACE KIT with TAP (Takara, Cat# 6017) according its guideline. The Special primers are Outer primer: Z-5 acggcggattgaccgtaatg, Inner primer: Z-2 tgtgagcgagtaacaacc. And other two primers, 5′ RACE Outer primer and 5′ RACE Inner primer were provided in the kits.

### Cell culture and transfection

SMMC7721 was cultured in RPMI 1640 medium, supplemented with 10% FBS, 100 units/mL penicillin and 100 ug/mL streptomycin and maintained in a humidified incubator with 5% CO_2_ at 37°C. Transfections were performed with Lipofectamine 2000 (Invitrogen, USA) according to the manufacturer's instructions.

### Fluorescence *in situ* hybridization

The LINC00052 biotin-RNA probe was synthesized with biotin-16-UTP (Roche, LOT 14687428) according to the procedure instructions of SP6 RNA Polymerase (Roche, LOT 12039672910). SMMC7721 cells were placed on slide and fixed 30 min at room temperature with 4% paraformaldehyde, then incubated 3 min at room temperature with 0.1% Triton-100. Blocking solution was used to incubate the cells 5 min at 42°C and replaced the Blocking solution with new Blocking solution, 30min at 42°C. Biotin-RNA probe was added to the Blocking solution in a final concentration 1ug/ml and incubated at 42°C 3 h. Then cells were washed with new Blocking solution and added Strepavidin-FITC (Abcam, ab136201) which was diluted at 1:300 and incubated at 42°C 2 h. After washing with Blocking solution 3 times, the DAPI (Beyotime, C1005) staining was done according to the procedure instructions.

### Plasmid construction

LINC00052 fragment was obtained by PCR, then the fragment was cloned into pcDNA3.1(+) vector and named as pcDNA3.1-LINC00052. The over expression vector of NTRK3 (pCMV-Sport6-NTRK3) was created by cloning the NTRK3 coding sequence into pCMV-Sport6 vector with the Kpn I/Xho I sites. The miR-128 and miR-485-3p fragments were amplified by PCR using the genomic DNA of SMMC7721 cells as a template. Then the amplified fragments were cloned into pTargetTM vector (Promega), named pTarget-128 and pTarget-485-3p respectively. The wild-type NTRK3 3′-UTR was amplified by PCR from genomic DNA as a template, and the PCR product was subcloned into pGL3-Control dual-luciferase miRNA target expression vector (Promega) immediately downstream of the luciferase gene, named pGL3-NTRK3 3′-UTR. All vectors constructed were confirmed by DNA sequencing. All primers are listed in Table 1.

**Table 1:** Primer sequences used for PCR or constructions of various plasmids

Amplifier primers	Primer sequence (5′–3′)
**Real-time PCR primer**	
Linc00052	F: CCTGAAGTTTCTCCATGAATTGTG R: GAGGGAGGGAGACTGAGATT
NTRK3	F: TGAGAACCCCCAGTACTTCC R: CTGAAACCATGTGACCTTGG
miR-128	TCACAGTGAACCGGTCTCTTT
miR-485-3p	GTCATACACGGCTCTCCTCTCT
U6	F: AGAGCCTGTGGTGTCCG R: CATCTTCAAAGCACTTCCCT
β-actin	F:GTGGATCAGCAAGCAGGAGT R: TGTGTGGACTTGGGAGAGGA
**Construction plasmid primer**	
NTRK3	F: CGGGGTACCATGG ATGTCTCTCTTTGCCC R: CCGCTCGAGCTAGCCAAGAATGTCCGGGT
miR-128	F:CCGCTCGAGATGTTAAACAGTCTCC R:CGGGGTACC ACATATTGTGTATATATTAC
miR-485-3p	F: CCGCTCGAGATGCGGCTTTGGGAAGC R: CGGGGTACCAAGATGCTTCTAGATGCCC
NTRK3 3′UTR	F:TGCTCTAGAACCCTTTAACACCACCAG R:TGCTCTAGAATGCTCTCCACCATTAGGTG

### Small interfering RNA synthesis

Small interfering RNA (siRNA) sequences against LINC00052 and NTRK3 gene were synthesized by Invitrogen. The siRNA sequences were as follows: LINC00052 siRNA1: 5′-UUAUUCACAUCACUGCAU GTT-3′, siRNA2: 5′-UUUCAGAUAUGCCAAGCUC TT-3′, a random scramble siRNA (NC) was used as a control: 5′-ACGUGACACGUUCGGAGAATT-3′. NTRK3 siRNA1:5′-UAA CAGCAUUGUCACCCUCTT-3′, siRNA2: 5′-AUUCCAAAUUUGGACCGUCTT- 3′, siRNA3: 5′-UUGGUAGUAUUCCACAUGGTT-3′, a random scramble siRNA (NC) was used as a control: 5′-ACGUGACACGUUCGGAGAATT-3′.

### Reverse-transcription reaction and quantitative real-time PCR

Total RNA was isolated with Trizol reagent (Invitrogen, USA). For detecting the expression of LINC00052 and NTRK3, the first-strand cDNA was generated with using the Reverse Transcription System (Promega, Madison, WI). For analysis the expression of miR-128 and miR-485-3p, the first-strand cDNA was generated with a miRNA cDNA Kit and a BioRT cDNA First Strand Synthesis Kit according to the manufacturer's instructions (Cwbio, China). Real-time PCR was performed to confirm the expressions of LINC00052, NTRK3, miR-128 and miR-485-3p. A cycle threshold (CT) was assigned at the beginning of the logarithmic phase of PCR amplification, and duplicate CT values were analyzed by the 2-∆∆ct method [32]. U6 snRNA and β-actin mRNA levels were used for normalization. All experiments were performed in triplicate and repeated at least 3 times.

### Stable cell generation

SMMC7721 cells were transfected with pcDNA3.1-LINC00052 and pcDNA3.1, then selected with G418 (1000 ug/ml). Two weeks later, few cells survived, and G418 was reduced to 500 ug/ml. Stable cell line pcDNA3.1-LINC00052, which could stable express LINC00052 was established, and expression was measured via Real time RCR.

### Transwell assays

For the transwell assays, 1 × 10^5^ cells suspended in 200 uL of serum-free RPMI 1640 were seeded into the transwell migration chambers (transwell membranes of 8mpore size, Costar). 800 uL conditioned RPMI 1640 medium with 5% (v/v) fetal bovine serum was placed in the bottom compartment of the chamber. After 36 h incubation at 37°C, the membrane was washed briefly with PBS, and the non-migrated cells on the upper surface of the membrane were removed with cotton swab. Migrated cells were fixed with 4% paraformaldehyde for 30 min, stained with 1% crystal purple for 1min, then washed with distilled water. The migrated cells were then counted using an inverted microscope after drying. All experiments were performed in triplicate and repeated at least 3 times.

### Wound healing assays

Cell migration was evaluated by wound healing experiment. Briefly, when cells grew in full monolayer on cover slips in six well plates, and cover slips were scraped a 2 mm channel by 200 ul tip and washed with PBS gently. Then the medium was immediately replaced with serum-free RPMI 1640. Phase contrast images were captured and marked 0 h. After 24 h, the cells were taken contrast images again. The width of wounds was measured in three-independent wound sites per group. Relative migration of cells was calculated with the healing distance. All experiments were performed in triplicate and repeated at least 3 times.

### Cell proliferation assay

Cells were trypsinized and seeded into 96-well culture plates with a density of 8000 cells/well. The cells were harvested at different time points (12, 24, 48, and 72 h) for growth assay using the MTS kit (Promega, USA) following the manufacturer's protocol and the absorption was read at 490 nm. All experiments were performed in triplicate and repeated at least 3 times.

### Dual-luciferase reporter gene assay

SMMC7721 cells were seeded at a density of 2 × 10^5^ cells/well in 24-well plates and co-transfected with 500ng pTarget-miR-128 or pTarget-miR-485-3p or 500ng pTarget plasmid, 300 ng pGL3-NTRK3-3′UTR plasmid or 300 ng pGL3-control plasmid and 50 ng of the control Renilla plasmid pRL-TK (Promega, USA) using Lipofectamine2000. A luciferase activity assay was performed 48 h after transfection using the dual-luciferase reporter assay system (Promega, USA). All transfection experiments were performed in triplicate and repeated at least 3 times.

### Western blot analysis

Total cell lysate was prepared in RIPA buffer supplemented with 1mmol/L PMSF. A BCA Protein Assay Kit (Beyotime, China) was used to determine the protein concentration. Equal amounts of total protein were separated by 10% SDS-PAGE and then transferred to a PVDF membrane. The membrane was blocked in 5% nonfat dry milk in TBST (Tris-HCl-buffered saline supplemented with 0.5 % Tween 20) for 2 h followed by primary antibody (rabbit anti-human NTRK3, diluted 1:2000; rabbit anti-human β-actin, diluted 1:5,000) overnight. The membrane was then incubated with secondary antibody conjugated with HRP (diluted 1:4,000) and visualized using an ECLTM chemiluminescence detection system (Pierce, USA). All experiments were performed in triplicate and repeated at least 3 times.

### Colony formation assay

Cells were seeded into a fresh 6-well plate and maintained for 2 weeks. Colonies were fixed with 4% paraformaldehyde and stained with crystal violet for 30 min, respectively, then photographed and analyzed. All experiments were performed in triplicate and repeated at least 3 times.

### Tumor growth assay

Female BALB/c nude mice (4–6 weeks old) were purchased from the Laboratory Animal Services Center of CUHK. Animal handling and experimental procedures were approved by the Animal Experimental Ethics Committee of CUHK. The four group cells: SMMC7721, A554, pc DNA3.1-LINC00052 and pcDNA3.1 with a total of 5 × 10^6^ cells respectively were injected subcutaneously (SC) into the hip back of nude mice. 4 weeks later, mice were sacrificed and tumors were dissected and weighed.

### Tumor migration assay

Female BALB/c nude mice (4–6 weeks old) were purchased from the Laboratory Animal Services Center of CUHK. The four group cells: SMMC7721, A554, pcDNA3.1-LINC00052 and pcDNA3.1 with a total of 5 × 10^6^ cells respectively were intrahepaticly injected. 24 days later, mice were sacrificed and tumor migrations were analyzed.

### Immunohistochemistry

Paraformaldehyde-fixed, paraffin-embedded tissue of transplanted tumors and human liver cancer tissue were sectioned at 4.5 μm thickness and analyzed for Ki-67 (Bioword, 1:100 dilution) and NTRK3 (Bioword, 1:200 dilution) expression. Visualization was achieved by using the 3, 3′-diaminobenzidine substrate. Sections stained with PBS only were used as the negative staining control.

### Statistical analysis

All data were expressed as the means and standard deviations. Differences between groups were assessed by X^2^ analysis and 2-tailed Student *t* test. The difference was deemed statistically significant at *P* ≤ 0.05.

## SUPPLEMENTARY MATERIALS FIGURES


