# Supplementary material for: LINC00052 regulates the expression of NTRK3 by miR-128 and miR-485-3p to strengthen HCC cells invasion and migration
Source: Oncotarget. 2016 Jun 23;7(30):47593–608. doi: 10.18632/oncotarget.10250 (PMC5216964; doi:10.18632/oncotarget.10250)
Supplement: Supplementary file 1 [file oncotarget-07-47593-s001.pdf]

LINC00052 regulates the expression of NTRK3 by miR-128 and miR-485-3p to strengthen HCC cells invasion and migration

Supplementary Materials

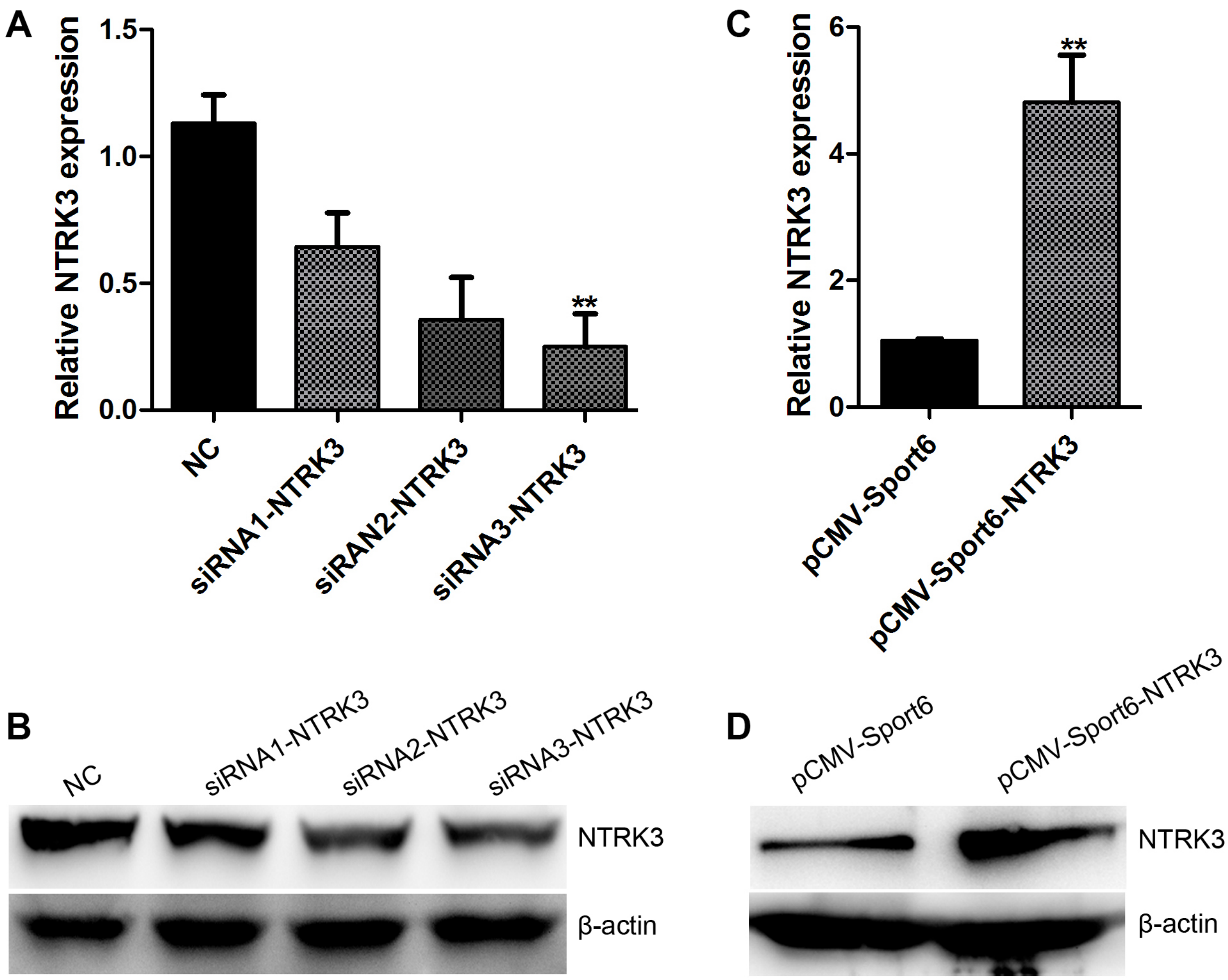

Supplementary Figure S1: Verification of over expression and siRNAs for NTRK3. (**A**), (**B**) Real-time PCR test and Western blot were used to identify siRNAs of NTRK3 and the siRNA3 has high interference efficiency. (**C**), (**D**) Real-time PCR test and Western blot were used to identify over-expression of NTRK3.

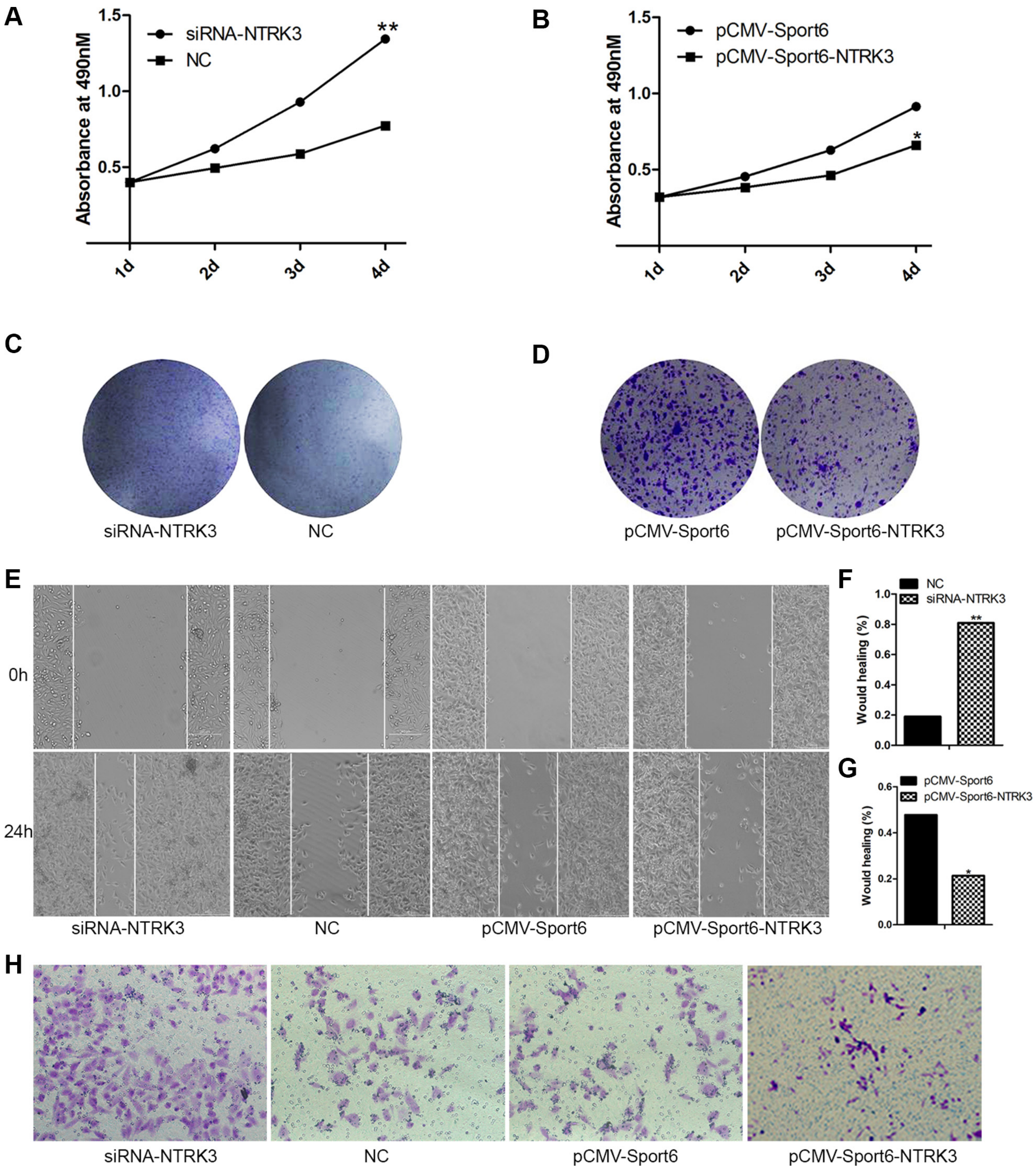

Supplementary Figure S2: NTRK3 regulated cells proliferation, invasion and migration in SK-Hep1 cells. The same experiments were performed as Figure 6.
